# T lymphocytes against solid malignancies: winning ways to defeat tumours

**DOI:** 10.15698/cst2018.07.148

**Published:** 2018-07-26

**Authors:** Ignazio Caruana, Luca Simula, Franco Locatelli, Silvia Campello

**Affiliations:** 1 Dept. of Pediatric Onco-Hematology and cell and gene therapy, IRCCS Bambino Gesù Children's Hospital, Rome, Italy.; 2 Dept. of Biology, University of Rome Tor Vergata, Rome, Italy.; 3 IRCCS, Santa Lucia Foundation, Rome, Italy.

**Keywords:** cancer immuno-therapy, immune cell infiltration, tumour microenvironment, extracellular matrix, immune-suppression

## Abstract

In the last decades, a novel field has emerged in the cure of cancer, by boosting the ability of the patient’s immune system to recognize and kill tumour cells. Although excellent and encouraging results, exploiting the effect of genetically modified T cells, have been obtained, it is now evident that tumour malignancies can evolve several mechanisms to escape such immune responses, thus continuing their growth in the body. These mechanisms are in part due to tumour cell metabolic or genetic alterations, which can render the target invisible to the immune system or can favour the generation of an extracellular milieu preventing immune cell infiltration or cytotoxicity. Such mechanisms may also involve the accumulation inside the tumour microenvironment of different immune-suppressive cell types, which further down-regulate the activity of cytotoxic immune cells either directly by interacting with them or indirectly by releasing suppressive molecules. In this review, we will first focus on describing several mechanisms by which tumour cells may dampen or abrogate the immune response inside the tumour microenvironment and, second, on current strategies that are adopted to cope with and possibly overcome such alterations, thus ameliorating the efficacy of the current-in-use anti-cancer immuno-therapies.

## INTRODUCTION

When a tumour mass starts growing in the body, T lymphocytes are activated inside secondary lymphoid organs (SLOs) and mount an immune response against immuno-stimulating tumour antigens presented by professional antigen presenting cells. This stimulation allows T lymphocytes to leave SLOs, thus reaching the tumour mass and eliminating malignant cells. Although the capability of the immune system to prevent tumour growth may last for years, tumour cells may eventually escape immune-surveillance 1 and create a chronic tumour microenvironment (TME), which predisposes to the generation of highly-inefficient, "dysfunctional" T cells, with impaired metabolic activity and cytotoxic functionality 2.

In this Review, we will focus on the mechanisms by which alterations in the architecture of the TME predispose to a dysfunctionality of T cells, once inside the tumour, thus limiting their ability to cope with a growing number of cancer cells.

## THE SEVERAL IMMUNE SUBTYPES INSIDE THE TME 

Most subtypes of the innate and adaptive immune cells infiltrate into a solid tumour mass and can be generally divided for their pro- or anti-tumor role (**Table 1**). Tumour-infiltrating T lymphocytes are commonly known as TILs. Cytotoxic CD8+ TILs are major player in the immune response against tumour growth and their recognition of tumour-specific antigens allows killing of malignant cells through Fas ligand or perforin/granzyme pathways. Usually, helper CD4+ TILs mainly produce several inflammatory cytokines, which can support the activation and cytotoxicity of CD8+ T cells and of other pro-inflammatory immune cells 3. However, a subgroup of CD4+ T cells, the regulatory T cells (Treg), play an anti-inflammatory and immune-suppressive role inside the TME, thus favouring tumour growth, by inhibiting dendritic cell (DC) presentation of tumour antigens to T cells and by producing membrane-bound or soluble factors, which impair T-cell activation and cytotoxicity 4. The role of tumour infiltrating B lymphocytes (TIBLs) in cancer progression is more debated. Although some reports indicate that TIBLs can have an anti-tumor effect, for example producing antibodies which favour tumour cell recognition by phagocytic macrophages, they can also differentiate towards a regulatory phenotype (Bregs), which produce pro-tumor factors, such as lymphotoxin (which promotes neo-angiogenesis) and immune suppressive cytokines 5.

**Table 1:** TABLE 1. Immune subtypes infiltrating solid tumors. List of innate and adaptive immune subtypes infiltrating solid tumour microenvironment. For each one, the pro- or anti-tumoral role (activity), the prevalent metabolism, and the main released factors -as soluble molecules or exposed on the cell surface, are reported. Abbreviations: TILs: tumor-infiltrating-T-lymphocytes; TIBLs: tumor-infiltrating-B-lymphocytes; DCs: dendritic cells; NK: natural Killer; ILCs: innate lymphoid cells (other than NK cells); MDSCs: myeloid-derived-suppressor-cells; TAM: tumor-associated-macrophages; IDO: Indoleamine-2,3-dioxygenase; iNOS: inducible Nitric Oxide Synthase; VEGF: vascular endothelial growth factor.

**Cell type**	**Activity**	**Metabolism**	**Released or surface factors ** (**pro-inflammatory / **anti-inflammatory)
**CD8+ TILs**	anti-tumoral	glycolytic	**IFNγ, TNFα, IL2, GranzymeB, Perforin**
**CD4+ TILS th1**	anti-tumoral	glycolytic	**IFNγ, TNFα, IL2**
**CD4+ TILS th2**	pro-tumoral	glycolytic	IL4, IL5, IL13, IL25, IL10
**CD4+ TILS th17**	anti-tumoral	glycolytic	**IL21, IL17a, IL17f, IL22**
**Tregs**	pro-tumoral	oxidative	IL10, TGFβ, IL35, CD39, CD73, IDO
**TIBLs**	anti-tumoral	mainly glycolytic	**IL2, IL4, TNFα, IL22, IL6, IL15**, GM-CSF
**Bregs**	pro-tumoral	oxidative (?)	Lymphotoxin, IL10, TGFβ, IL12, PDL1, FasL
**DCs**	anti-tumoral	glycolytic	**IL2, IL12, IFNγ, TNFα**, IL10, TGFβ, IL6, IL4
**NK cells**	anti-tumoral	mainly glycolytic	**IFNγ, TNFα, IL2, GranzymeB, Perforin**
**ILCs**	mixed	mixed	ILC-1, -2, -3 similar to th-1, -2 and -17 subsets
**MDCs**	pro-tumoral	oxidative	Arginase, PGE2, IDO, TGFβ, M-CSF
**TAM M1-like**	anti-tumoral	glycolytic	**IL6, IL12, IL23, TNFα, Cxcl5, Cxcl9, Cxcl10**, IL1β, iNOS
**TAM M2-like**	pro-tumoral	oxidative	IL10, TGFβ, IL6, Arginase, VEGF, IL1β

DCs are required to uptake and present tumour-derived antigens to adaptive T cells into secondary lymphoid organs (SLOs). Therefore, their infiltration into tumour mass is required to mount an efficient adaptive immune response against tumours, and to sustain the generation of tumour-specific T lymphocytes for an efficient elimination of malignant cells 6. Innate lymphoid cells (ILCs) are a large family of innate cells whose subdivision is very similar to that of T lymphocytes, although they lack antigen-specific receptors and are mainly activated in response to common innate signals. Indeed, among them, we can find cytotoxic natural killer (NK) cells (equivalent of CD8+ T lymphocytes) and helper-like ILCs, which further subdivide into ILC-1, -2, and -3 (which are functionally similar to Th1, Th2 and Th17 CD4+ T cells, respectively) 7. NK cells are cytotoxic cells which can recognize and kill tumour cells that lack the expression of major-histocompatibility-complex (MHC) molecules, and therefore cannot present antigens on cell surface, this rendering them invisible to cytotoxic CD8+ T cells 8. Between regular α/β T lymphocytes and NK cells, a subgroup of T cells named γ/δ T lymphocytes combine both innate and adaptive characteristics. Their potent cytotoxic activity against bacteria, virus and tumours makes them particularly attractive for adoptive immunotherapy approaches. Differently from αβ T cells, these cells recognize their ligands in an MHC-independent manner showing a significant reduced allo-reactivity as compared to αβ T cells, this making them appealing for clinical translation 9,10. Contrary to NK cells and γ/δ T lymphocytes, the role of other ILCs in cancer has only recently been discovered and few related data are available. However, it seems that ILC2 and ILC3 subtypes may favour tumour growth by secretion of specific immune-suppressive cytokines 7. Similarly, myeloid-derived-suppressor-cells (MDSCs) are pro-tumoral cells, producing factors that inhibit T-cell activity and also promote tumour growth by remodelling extracellular matrix and blood capillaries 11. Last, macrophages can differentiate into different subtypes inside the TME, where they are known as tumor-associated macrophages (TAM). Briefly, an anti-tumor M1-type TAM can produce inflammatory cytokines and phagocyte tumour cells 12. However, most TAMs have a pro-tumor M2-like phenotype, favouring tissue remodelling and angiogenesis and producing immune suppressive cytokines, which dampen T cell response 12.

It is worth noting that the amount and subtypes of immune cells present in TME may vary widely between different tumours. This heterogeneity has led to the development of a classification into "immune-desert", "immune-inflamed" and "immune-excluded" tumours 13, based on the quality and quantity of immune infiltrates. Immune-desert tumours show poor infiltration of T cells and are characterized by high number of myeloid suppressor cells, which also produce immune-suppressive cytokines. Immune-excluded tumours are characterized by a high amount of infiltrating immune cells, both immune-suppressive (pro-tumor) or cytotoxic (anti-tumor). However, these cells do not frequently penetrate inside the tumour parenchyma but remain in the surrounding stroma and therefore cannot efficiently kill malignant cells. Last, immune-inflamed tumors are characterized by high amounts of infiltrating T cells, which efficiently reach tumour cells for their killing, and by abundant production of pro-inflammatory cytokines 13.

## THE FIRST SIDE OF THE COIN: TME IN SOLID MALIGNANCIES

Tumours have been recognized as complex disorganized and chaotic organs, where cancer cells co-exist and co-evolve with their stroma. The interface between malignant and non-transformed cells defines the TME 1,14. The importance of TME for tumorigenesis is now widely recognized in both solid and haematological malignancies 15. However, in solid tumours, the TME has a more relevant impact on tumour growth being able to offer protection with respect of the action of the immune system. During solid tumour development, the TME initiates to organize itself supporting tumour growth directly, but also erects chemical and physical barriers capable to defend the tumour from the activity of an intact immune system, thereby preventing cancer immune surveillance. In this context, TME appears to be a complex ecosystem containing a tight interstitial extracellular matrix (ECM), where various stromal, endothelial and inflammatory cells are recruited from the surrounding tissues. The interaction between these different components modulates phenotype and behaviour of the tumour and may affect cancer progression, as well as the formation of metastases 16,17,18. Specifically, it has been reported that, in this environment, cancer cells show self-sufficiency in growth signals, resistance to programmed cell death, limitless replicative potential, and ability to induce angiogenesis, invasion, and metastasis formation 19,20. Recently, the role of ECM in the regulation of many of these cellular responses has been recognized. ECM has a fundamental role in cell behaviour and fate, not only sustaining and interconnecting cells, but also influencing many cellular mechanics and functions, such as differentiation and migration, in both physiological and pathological conditions.

Cancer cells are also able to develop mechanisms to blunt detection and eradication by immune cells. These strategies include: i) a reduction of tumour immunogenicity, due to loss of expression of tumour-associated antigens or MHC class I molecules, ii) acquired DNA copy number alterations and oncogenic signaling -equipping them with an uncontrolled proliferative capacity and insensitivity to negative feedback from microenvironment, iii) an up-regulation of cellular immune check-points - such as the programmed death ligand 1 (PDL1, which inhibits T-cell activation by stimulating PD-1 receptor on T-cell surface), indoleamine 2,3-dioxygenase, and finally iv) an altered metabolism producing a low pH and secretion of various metabolites, which inhibit the effector cell recruitment, persistence and activity 21,22,23,24. In particular, tumour cells can evolve mechanisms which actively induce T-cell apoptosis, by up-regulating the expression of pro-apoptotic molecules on their surface, such as Galectin-1 25, TRAIL (TNF-Related Apoptosis-Inducing Ligand) 26 and Fas Ligand (FasL) 27, which promote T-cell death by interacting with the corresponding receptors on T-cell surface. Moreover, in the TME, a competition for nutrient availability is frequently observed between tumour and immune cells. Although cancer stem cells may rely on an oxidative metabolism for their survival 28, in most cases, tumour cells switch their metabolism from oxidative phosphorylation (OXPHOS) to glycolysis even in presence of high-oxygen tension to sustain a high proliferation rate (a process known as Warburg effect 29). Since this switch is also observed for T-cell upon activation 30, this generates a competition for glucose availability between these cell types. However, tumour cells frequently win the battle probably because of a faster glycolytic rate in tumour cells 31 and a further down-regulation of glycolytic flux in immune cells by tumour cells-released extracellular lactate 32,33. As mentioned above, tumour cells may up-regulate PDL1 on their surface 34,35, which engages the PD-1 receptor on activated T lymphocytes, thereby activating a signalling cascade that inhibits PI3K/Akt/mTOR axis 36, essential to induce glycolysis in effector T cells. Interestingly, while these considerations apply to effector cytotoxic T cells, immune Tregs actively maintain an OXPHOS-based catabolism instead of glycolysis 37,38. This confers to Tregs a metabolic advantage compared to effector/cytotoxic T cells 39. To further increase the suppressive TME, several other molecules are secreted by tumour cells and by other immune-cells recruited by the tumour. For example, in a preclinical study it has been reported how the secretion of prostaglandin-E2 (PGE2) and adenosine by endothelial tumour-associated cells selectively kills effector T cells and how their inhibition resulted in down-regulation of FasL and CD8 T-cell influx 40.

Besides Tregs, other cells of the immune system are recruited to the TME as MDSC, TAMs and neutrophils. All these cells boost the tumour survival-promoting environment 41,42,43,44 i) by reducing, for example, L-arginine concentration, which is required for long-lasting survival of infiltrating memory T cells 45, ii) by producing reactive nitrogen species that hamper T-cell proliferation and function, and iii) by expressing molecules on their surface capable to activate check-point inhibitor receptors expressed by T lymphocytes (Galactin9) 44,45,46,47. The described TME with all these elements is able to regenerate and stabilize itself.

Making matters worse, tumour cells and their TME are able to initiate and promote angiogenesis. This phenomenon induces the formation of new vessels capable to support blood supply to the tumour; the resulting vessel network is leaky, chaotically organized, immature, thin-walled and ill-perfused. Such an aberrant angiogenesis contributes to the maintenance of the pro-tumorigenic and immunosuppressive TME and profoundly influences how cancer cells escape the anti-cancer immune surveillance, metastasize, and respond to immunotherapy 48,49,50,51. This occurs by preventing, for instance, a correct inflow of the immune system to the tumour site. Furthermore, this reduced influx of nutrients and gaseous exchange strongly decreases the quality and number of effector cells in the TME, thus increasing tumour growth as well as the possibility of invasiveness of tumour cells 52,53. For these reasons, the TME represents an inhospitable and inaccessible environment for effector immune cells, due to the generation of a hypoxic atmosphere, low nutrient supply and a high concentration of metabolic acids. These conditions facilitate the selection of cancer cells with genetic and epigenetic alterations, which enhance their aggressiveness. In the meanwhile, they increase activation-induced autophagy processes and stress in immune cells, which makes cytotoxic lymphocytes unable to proliferate and produce cytokines 54,55. Hypoxia in the TME can dampen T-cell functionality through different mechanisms, such as: i) by exacerbating glucose deprivation, ii) by reducing cytosolic levels of Ca^2+^, which is essential for cytokine production 56, or iii) by promoting excessive formation of reactive oxygen species 57. In addition, it has been proven that hypoxia can up-regulate PDL1 on tumour cells 58, which in turn dampens T-cell functionality by interacting with the inhibitory receptor PD1 on T-cell surface. Importantly, hypoxia and acidosis, besides reducing the cytotoxic activity of tumour-infiltrating effector T cells, also facilitate the attraction and/or development of immuno-suppressive immune cells, and hamper delivery of chemotherapeutics and immunotherapeutic entities, as well as cancer cell killing in response to radio/chemotherapy and immunotherapy.

The recruitment of particular cell types into TME and their contact with tumour cells has been described to produce an immuno-suppressive microenvironment, for example, due to the secretion of PGE2 and adenosine.

To further protect itself, the tumour establishes strong interactions with the corrupted stromal cells, by also initiating the production of a physical barrier remodelling the ECM. This is achieved, for example, by the modification of soluble factors (cytokines, growth factors, hormones), type of cells, and structural proteins (collagens, laminins, fibronectins, proteoglycans and hyaluronans), with the latter altering the normal stiffness and adhesion strength of the ECM 59,60,61. In summary, these biomechanical changes involve not only cancer cells but also their ECM and the entire TME components. The increase of ECM stiffness, for instance, promotes cancer invasion and progression 62. Moreover, the recruitment of other cells including fibroblasts, myofibroblasts, granulocytes, macrophages, mesenchymal stem cells and lymphocytes in the surrounding stroma, could also be responsible for the hard consistency of tumours at a macroscopic scale. Also, cancer-associated fibroblasts (CAFs) reorganize the stroma by secreting new ECM elements and enzymes that covalently cross-link collagen fibres and pull the collagen network closer together.

## THE OPPOSITE FACE OF THE COIN: THE STRESS OF T LYPMPHOCYTES IN THE ATTEMPT TO SURVIVE, REACH AND KILL TUMOUR CELLS

An efficient T-cell response depends on several aspects related to both tumour cells, as described, as well as on factors associated with T-cell activation and functionality. In order to be effective, T lymphocytes require three signals: i) the interaction of the antigenic peptide-MHC complex with the T-cell receptor (TCR), ii) the binding with the co-stimulatory or co-inhibitory ligand, provided by antigen-presenting cells, and iii) the stimulation/proliferation mediated by extracellular cytokines such as interleukin (IL)-2 and IL-15 63. Among these signals, the second one determines the promotion or inhibition of T-cell cytokine production and effector function; appropriate co-inhibitory signals dampen inflammation to avoid tissue damage due to an excessive immune reaction, whereas durative and excessive co-inhibitory signals lead to T-cell hypo-responsiveness 64. Then, in many cases, tumour antigens are weakly immunogenic self-molecules and most tumour-specific T cells have low precursor frequencies and low TCR affinity. This phenomenon occurs because T cells with high avidity against self-molecules, including also tumour self-antigens, are normally deleted during thymic T cell education 65. In addition, it has been proven that the antigen presentation process is strongly impaired in TME, this leading to insufficient priming and boosting of T lymphocytes 66. As mentioned above, down-regulation of MHC proteins makes tumour cells "invisible" to infiltrating effector T cells, this resulting into a dysfunction of their anti-tumour activity. This effect is further enhanced by a lack of costimulatory molecules in several solid and haematopoietic tumours 67.

The presence of anti-inflammatory soluble factors released by tumour-associated corrupted cells, such as IL-10, transforming-growth-factor-β (TGFβ), cyclooxygenase-2 (COX2), inducible nitric oxide synthase (iNOS) and PGE2, induces the expression of several negative ligands (FasL, PDL1, PDL2, Galactin 9, ect.) on the cells present inside the TME, including T lymphocytes, whose response is therefore inhibited (**Table 2**). T lymphocytes infiltrating the TME may thus undergo functional exhaustion. This "exhausted" signature is progressively acquired with time, mainly due to a continuous stimulation of the TCR, followed by a progressive increase of expression of co-inhibitory receptors (PD-1, LAG-3, TIM3, CTLA-4, BTLA and TIGIT) 68,69,70,71, and by a decrease of cytokine production and proliferation potential. All these modifications make exhausted T cells unable to differentiate back into functional memory cells, even if antigen stimulation is removed 72. Interestingly, this "exhausted" state is hard-wired to epigenetic modifications into the T cell genome. Given the frequent chronic nature of a cancer, it is not surprising that exhausted T cells have been found in several tumours 73,74,75. In this scenario, also TAMs, MDSC 76 and Treg 77 facilitate the generation of exhausted cells in the TME 78. Also, the fine-tuning control of ionic balance in the TME is an additional check-point for an efficient T-cell functionality. Indeed, potassium ions, released by necrotic cells in the extracellular milieu, can be internalized by infiltrating T cells, thus inhibiting their effector functions by downregulation of the Akt/mTOR signalling, downstream of TCR stimulation 79. Similarly, tumour-infiltrating human T cells expressing high levels of the calcium channel Kv1.3 can sustain their calcium influx upon TCR stimulation also in environment with low-calcium levels, thus improving their cytotoxicity against tumour cells 80. The presence of an immune-suppressive environment and the absence of adequate chemotactic factors significantly reduce recruitment of new immune cells from the periphery or from lymphatic organs. To migrate towards a particular tissue, immune cells need the presence of a particular environment produced by cytokines or chemokines, these creating the *in situ* correct attractive chemical gradient. Every TME produces a specific offset of cytokines and chemokines capable to attract or repel different cell types. Chemokine gene expression profiles and immune cell infiltration have been investigated in different tumour types 81,82,83. To hinder T cell migration to the tumour site, tumour-derived chemokines may misdirect activated T cells to the tumour surrounding stromal cells 84, and cancer cells can further post-transcriptionally modify their chemokine expression profile. For example, CCL2 nitrosylation can reduce its chemoattractive effect on effector T cells, but not on MDSCs 85.

**Table 2:** TABLE 2. Factors regulating CD8+ TIL functionality inside the TME. List of molecules that can inhibit, or sustain, tumour-infiltrating-T-lymphocyte (TIL) functionality inside the tumour microenvironment. The corresponding receptors on TIL surface of the indicated check-point molecules are reported in brackets. Abbreviations: TILs: tumor-infiltrating-T-lymphocytes; IDO: Indoleamine-2,3-dioxygenase; COX2: cyclo-oxigenase2; PGE2: Prostaglandin E2.

**TILs inhibitory factors**	
Soluble cytokines	IL-10, TGFβ (*from myeloid cells and Tregs*)
Inhibitory Check-point	PD-L1/2 (*from myeloid and tumor cells, bind *PD-1), B7-H4 (*unknown target*), CD276 (*binds CTLA4*)
Metabolites	Oxide, IDO, COX2 metabolites, PGE2 (*from myeloid and tumor cells*), adenosine (*from myeloid cells and Tregs*)
**TILs co-stimulatory factors**	
Soluble cytokines	IL2, IL7, IL15, IFNγ, TNFα (*from pro-inflammatory myeloid and lymphoid cells*)
Stimulatory Check-point	CD86/80 (*bind CD28*), CD70 (*binds CD27*), CD137L (*binds *CD137), CD235 (*binds CD134*), CD58 (*binds *CD2), B7 (*binds CD28*), OX40L (*binds OX40*), 4-1BBL (*binds 4-1BB*)
Metabolites	arginine (*memory survival*), fatty acids (*oxidative metabolism*)

Furthermore, the presence of a compact ECM increases the incapacity of T lymphocytes to recognise and kill tumour cells, since T lymphocytes need to actively infiltrate the TME to reach the neoplastic cells. Invasion is an active process in which secretion of particular enzymes is needed to degrade the ECM elements. Several groups, including our own one, reported how this invasion capacity is reduced in patients after high dose chemotherapy and after adoptive T cell transfer, in both cell and gene therapy settings 86. In the last years, different researches focused on the optimization of chemotherapy regimens in combination with immunotherapies 87,88, such as Treg depletion 89,90, blockage of check-point inhibitors 91,92, anti-angiogenesis treatment 93,94, use of oncolytic virotherapy 95,96 and gene modification of T lymphocytes 86,97, in order to improve the penetration of T lymphocytes or other effector cells. This aspect will be addressed in the following paragraph.

## PRE-CLINICAL AND CLINICAL DATA ON REDUCING T-CELL STRESS AND IMPROVING ANTI-TUMOUR ACTIVITY

In the last two to three decades, the development of a new field of research has focused on advanced anti-cancer immunotherapies. Strategies include monoclonal antibodies, adoptive cell transfer, check-point inhibitors, vaccine therapies, oncolytic therapies and gene modification of effector cells, all these approaches showing remarkable long-term efficacy in patients with various types of cancers 98,99,100,101,102,103. Although conventional therapies, such as radiation and chemotherapy, induce positive responses in the majority of patients, relapse and resistance often occur in patients after prolonged treatment 104. The strategies developed so far have focused their attention on implementation of tumour targeting. Recently, several pre-clinical and clinical studies have highlighted how most of the pitfalls observed during cancer treatment, in particular in the setting of solid tumours, are not only related to the presence of heterogeneous subpopulations of cancer cells, but also to the development and/or recruitment during tumorigenesis of particular cell types and chemical-physical barriers, which are instrumental to the pathologic manifestation of cancer 105,106.

Clinical trials underline how the presence of adequate and functional T cells within tumours correlates with favourable clinical outcome. Several studies in animal models have been carried out in order to fully understand how T-cell infiltration can be enhanced to promote tumour rejection, or to prevent recurrence 107,108,109. The ever-deeper knowledge of the TME and its constitutive elements, and development of multidisciplinary approaches are laying the foundations for technologies that, one step at a time, are trying to tackle the problem of improving the invasive capacity of T lymphocytes, while improving both their ability to survive and their tumour specificity.

Immunotherapies targeted to counteract the mechanisms of tumour-induced T-cell dysfunction have successfully provided persistent clinical benefits in patients with advanced cancer. Most recently, they have focused on immune check-point blockade in order to block the activation of co-inhibitory receptors, or to reduce their levels on the surface of exhausted T cells in cancer patients. Most of these therapies proved to be successful by increasing T-cell functionality inside the TME by restoring their cytokine production and/or cytotoxic activity. Up to now, several check-point inhibitors are currently tested in clinical trials 110 including: combinations of PD1 or of its ligand PDL1, CTLA4 and LAG3 signalling inhibitors 100,101,111,112,113, or agents promoting the immune response with CD40/CD40L, CD137, OX40 and GITR stimulation/engagement 114.

Up to now several pre-clinical and clinical studies have shown the feasibility to redirect T lymphocytes on cancer cells through chimeric antigen receptors (CARs) in order to guarantee the specificity of T lymphocytes, overcoming the problem of human MHC protein down-regulation and the lack of costimulatory molecules 115. A typical CAR consists of a single-chain variable fragment (scFv) linked to an intracellular signalling domain, derived normally from T cells, and more recently also from NK cells 116,117. A more advanced generation of these molecules is being engineered to recapitulate the costimulatory events that occur upon TCR triggering to fully activate T lymphocytes. Signaling domains derived from T-cell costimulatory receptors are thus directly incorporated in tandem with the TCR co-receptor CD3ζ chain. Intra-cytoplasmic signaling domains of CD28, CD134 (OX40), CD137 (4-1BB), inducible costimulator (ICOS), CD27, DAP10 or CD244 (2B4) in various combinations have been used to construct second and third-generation CARs 118. After binding to tumour antigen through the scFv, the CAR activates T cells in an antigen-specific and MHC-independent manner, inducing lysis of the engaged target cells through granzyme-B and perforin pathways. Clinical trials with CAR T therapy have shown incredible efficacy in patients with acute lymphoblastic leukaemia, with a complete response rate of nearly 90% un patients who had already failed several lines of conventional therapies, including allogeneic hematopoietic stem cell transplantation 119. However, attempts to apply CAR T therapy to solid tumours has been less successful 120, and extensive efforts have been devoted to increasing CAR T-cell activity inside solid tumours, as well as their target specificity. For example, engineering CAR T cell to produce cytokines such as IL-7 and CCL19 have proved to increase their infiltration into solid tumour mass, leading to complete regression of pre-established tumours and prolonging survival in mice 121.

Several pre-clinical studies brought forward new strategies that can be applied to increase the infiltration of T cells, taking advantage of the negative feedback present in the TME, and producing particular factors capable to boost their persistence, as well as recruiting the innate immune system and inflammatory components. We proved that the T lymphocytes overexpression of heparanase, one of the enzymes involved in the ECM modelling, significantly increased the capacity to degrade the ECM, thus resulting in enhanced tumour infiltration and antitumor activity 86. Another updated strategy to implement T-cell infiltration is that based on the use of oncolytic viruses, which are able to infect and kill only tumour cells. Unfortunately, although highly promising *in vitro* 107, it did not show the expected results in clinical trials. Indeed, our group proposed the possibility to further arm the oncolytic therapy with chemokines and cytokines and to combine it with a CAR T-cell approach. The results of this strategy underline how this combinatory therapy is able to improve significantly tumour eradication and T-cell persistence 96. Based on this data, a growing number of clinical trials proposed to evaluate the clinical efficacy of: check-point inhibitors of soluble mediators (IDO, A2aR, CSF1R, IL-10 or TGFβ), agonistic antibodies targeting and activating receptors on T cells, anti-tumour vaccines 122,123 and adoptive transfer of CAR T cells 124,125.

**Figure 1:**
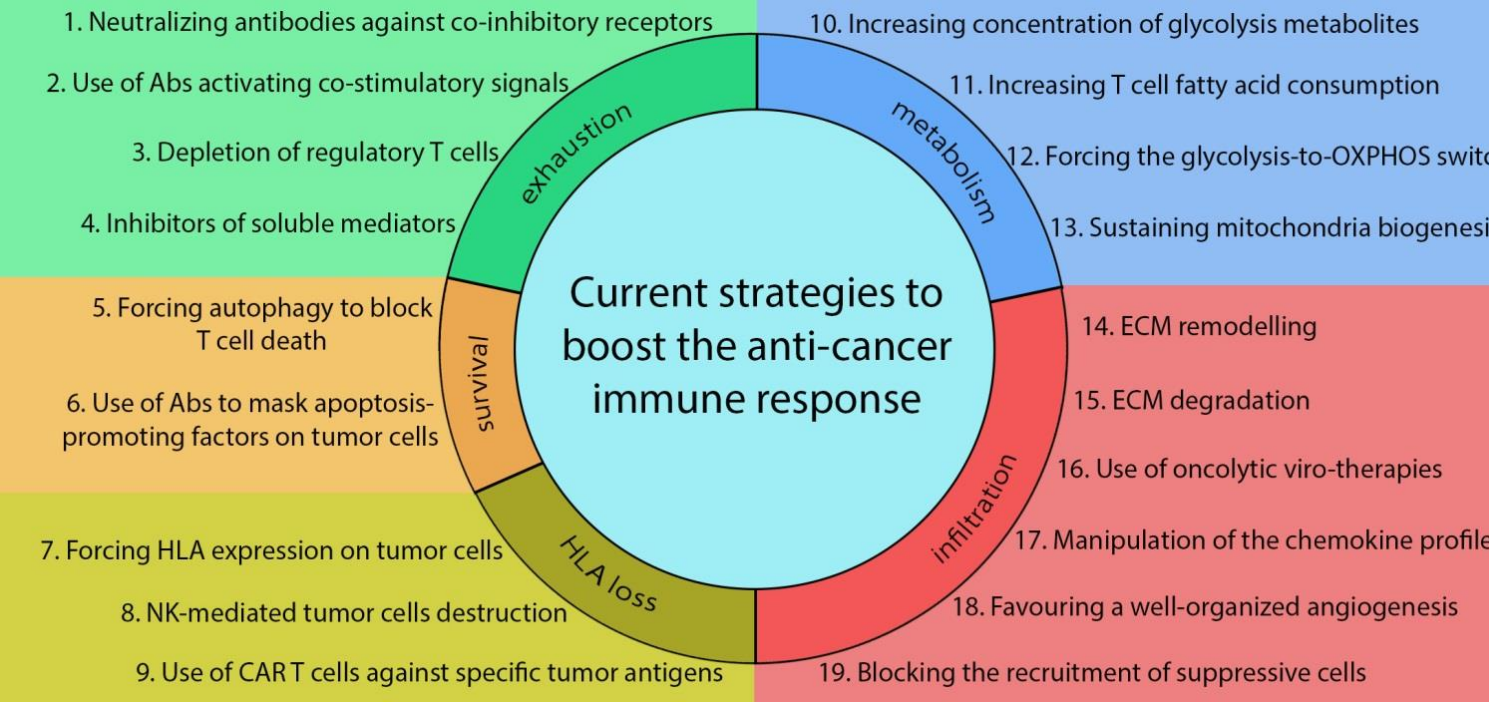
FIGURE 1: Current strategies to increase anti-cancer immune responses. The panel summarizes current strategies adopted in clinical trials or in basic research to increase the efficacy of immune-therapies against solid malignancies. Acting on T-cell exhaustion, researchers are currently testing the possibility to use soluble antibodies to neutralize co-inhibitory receptors (1) or to activate co-stimulatory signals on T cells (2). Other approaches involve depletion of immune suppressive Tregs (3) or the inhibition of soluble mediators produced by immune suppressive populations (4). Moreover, strategies could be adopted to prevent T-cell death inside the TME, by forcing autophagy in T cells (5) or by inhibiting the interaction between apoptosis-promoting factors on the tumour cell surface with apoptosis receptors on T cells using soluble neutralizing antibodies (6). In those cases, in which tumor cells evade the immune response due to a lack of antigen presentation, it is possible to force HLA expression on tumor cells (7) or to exploit the ability of NK cells to recognize and kill HLA-negative cells (8). Very recently, engineered CAR T cells have been used for their ability to kill tumour cells expressing specific antigens (10). Moreover, T cell functionality inside the TME may be increased through the modulation of its metabolism by increasing glycolysis metabolite concentration in the TME (10), by forcing T cells to utilize alternative substrates, such as fatty acids (11) or OXPHOS substrates (12), or by directly sustaining their mitochondria biogenesis and oxidative metabolism (13). Finally, several approaches are currently adopted to increase T-cell infiltration into the TME by forcing the expression of specific molecules by T cells, which can thus remodel (14) or completely degrade (15) the extracellular matrix (ECM), also by means of oncolytic-viruses (16). In addition, T-cell infiltration can be increased by modifying the chemokines expressed into the TME (17) or favouring a well-organized angiogenesis (18), which increases the ability of T cells to invade the tumour-surrounding stroma. By contrast, modulation of the chemokine profile could be used to prevent recruitment of suppressive immune populations into the TME (19). See text for details.

Furthermore, different groups investigate also the possibility to manipulate the TME chemokine profile in order to recruit sufficient numbers of effector cells into the tumour sites. In this regard, interesting data were produced with T,cell chemo-attractants, such as CCL4, CCL5, CCL21, CXCL10, TNFα, IFNβ and TNFSF14 96,126,127,128,129. Nevertheless, alternative strategies increasing T cell infiltration into the tumour mass is one of the main challenges that researchers will still have to face in the future. In this way, unconventional and unexpected regulators of these processes, such as mitochondria-dependent myosin fuelling of T-cell migration (see next paragraph for further details) 130,131, could unmask additional therapeutic opportunities to be exploited. In addition, new strategies, to be developed in future, could consider acting on satisfying the metabolic requirements of T cells inside the TME, because of the frequent low nutrient availability and hypoxic conditions. According to this hypothesis, several encouraging results have been recently obtained, such as for example, forcing T cells to use metabolites alternative to glucose, as fatty acids 132 (**Fig. 1**).

In recent years, several studies have highlighted the role of mitochondria in regulating several key processes in T cells. It has long been recognized that, while effector T cells upregulate glycolysis to quickly produce adenosine triphosphate and to generate precursors for biosynthesis of macromolecules 133, memory T cells mainly rely on mitochondria-based oxidative metabolism, sustained at least in part by fatty acid oxidation 134. Interestingly, the morphology of the mitochondrial network is tightly linked to the cell metabolic status and it can actively control it. Indeed, pharmacological manipulations favouring mitochondria elongation and OXPHOS activity, on *in vitro* isolated T cells, have been shown to reprogram T cells towards a memory phenotype, thus favouring their long-term survival and increasing their anti-tumour function 135. In addition, forcing T cells to utilize alternative pathways, instead of glycolysis, may favour their survival in glucose-deprived TME. In this way, increasing mitochondria-based fatty acid utilization could increase T-cell functionality inside the tumour 132. In addition, a recent paper shows that TILs undergo downregulation of mitochondrial mass inside the TME 136. Interestingly, the chronic antigen stimulation inside the TME leads to upregulated Akt levels, which, in turns, repress the activity of peroxisome proliferator-activated receptor gamma coactivator 1α, (PGC-1α) the master regulator of mitochondrial biogenesis. Therefore, dysregulation of mitochondrial oxidative metabolism, shut-down by hypoxia, has a strong negative effect on TILs functionality 136. Besides regulating T-cell metabolism, mitochondria have also been implicated in T-cell migration, proliferation and apoptosis, all key aspects for an optimal T-cell anti-tumour response. We demonstrated that dynamin-related protein 1 (Drp1)-dependent mitochondria remodelling is crucial to sustain T-cell chemotaxis 130 and we have clues that it also controls extravasation towards a solid tumour mass. Moreover, Drp1-mediated fission of mitochondrial network is essential for the redistribution of these organelles to daughter cells during cell division 137 and, in the absence of this process, the clonal expansion of T cells upon activation is strongly impaired (*unpublished personal results*). Therefore, modulation of mitochondrial dynamics may represent an important tool, in the future, to increase T-cell invasiveness and expansion into solid malignancies, in addition to modulate their energy utilization. This could become highly interesting, particularly for those "immune-excluded" tumours, where immune cells do not efficiently infiltrate into the tumour mass.

Mitochondria are also responsible for the release of cytochrome-C (cyt-C) in the cytosol during apoptosis. Although this process has long been investigated in cancer cells 138, we recently demonstrated that the morphology of the mitochondrial network tightly regulates also the physiological T-cell Activation-Induced Cell Death (AICD), a process normally involved in the shut-down of the immune response and exploited by tumours to kill them in the TME 139, by promoting cyt-C release 140. Indeed, the high rate of effector T-cell apoptosis inside the TME, due to chronic antigen stimulation, is one of the main obstacles for an efficient immune-response against solid malignancies. Again, engineering *in vitro* manipulated T cells to overcome this mitochondria-based processes could represent a novel approach to increase T-cell survival in the TME, and promising results have been recently obtained in this direction 141,142. At last, during T-cell stimulation, the autophagic machinery has important roles for an optimal T cell functionality. For example, it is essential to sustain T-cell survival and proliferation and it also controls the generation of long-lived memory T cells (see 143 for a review). Interestingly, we recently demonstrated that autophagy inhibition is necessary and strictly regulated to allow the onset of AICD, while forcing its activation prevents T-cell death 140. Whether modulation of autophagy could be exploited to overcome the high rate of apoptosis in TME infiltrating T cells is a still an unexplored field, but it may represent another promising tool for future therapeutic purposes.

Overall, this large mass of data suggests that additional and completely new targets in T cells have been unmasked in crucial cellular processes and organelles, which could be exploited in the future to boost anti-cancer T cell response.

## Abbreviations

CAR: chimeric antigen receptor
DC: dendritic cell
ECM: extracellular matrix
ILC: innate lymphoid cell
MDSC: myeloid-derived suppressor cell
MHC: major-histocompatibility complex
NK: natural killer
OXPHOS: oxidative phosphorylation
SLO: secondary lymphoid organ
TAMa: tumour-associated macrophage
TCR: T-cell receptor
TIL: tumour infiltrating T lymphocytes
TME: tumour microenvironment
Treg: regulatory T-cell
